# Postoperative Recovery of Balance Function in Lumbar Spinal Stenosis: A 12-Month Longitudinal Study Using the Brief BESTest and Its Association with Patient-Reported Outcomes

**DOI:** 10.3390/jcm14155520

**Published:** 2025-08-05

**Authors:** Tomoyoshi Sakaguchi, Masato Tanaka, Shinya Arataki, Tadashi Komatsubara, Akiyoshi Miyamoto, Mandar Borde, Umarani Arvind, Kazuhiko Takamatsu, Yosuke Yasuda, Adrian Doană-Prodan, Kaoruko Ishihara

**Affiliations:** 1 Department of Rehabilitation, Okayama Rosai Hospital, 1-10-25 Chikko Midorimachi, Minami-ku, Okayama 702-8055, Japan; tomoyoshi0127@gmail.com (T.S.); kazuhikopt0803@gmail.com (K.T.); kyushudanji19861007@gmail.com (Y.Y.); adrianoprodan@gmail.com (A.D.-P.); kaoruko0131@gmail.com (K.I.); 2 Department of Orthopaedic Surgery, Okayama Rosai Hospital, Okayama 702-8055, Japan; araoyc@gmail.com (S.A.); t.komatsubara1982@gmail.com (T.K.); hello.akkun.11.136@icloud.com (A.M.); mandarborde@gmail.com (M.B.); arvindumarani94@gmail.com (U.A.)

**Keywords:** lumbar spinal stenosis, Balance Evaluation Systems Test, balance function, Oswestry Disability Index, Modified Falls Efficacy Scale, Zurich Claudication Questionnaire

## Abstract

**Study Design:** Prospective observational study. **Background:** Lumbar spinal stenosis (LSS) impairs balance and gait function, increasing fall risk and limiting quality of life. Although postoperative recovery of balance is clinically important, longitudinal data using multidimensional balance assessments are limited. **Methods:** A prospective cohort study was conducted in 101 patients (mean age 74.9 ± 6.9 years) undergoing surgery for LSS. The Brief Balance Evaluation Systems Test (Brief BESTest), Oswestry Disability Index (ODI), Modified Falls Efficacy Scale (MFES), Zurich Claudication Questionnaire (ZCQ), and Visual Analog Scales (VAS) for pain/numbness were evaluated preoperatively and at 6 and 12 months postoperatively. Changes over time and correlations between Brief BESTest and PROMs were analyzed. **Results:** The total Brief BESTest score significantly improved from 13.3 ± 5.3 preoperatively to 16.1 ± 5.1 at 6 months and 16.0 ± 5.1 at 12 months (*p* < 0.01). Subdomains including Anticipatory Adjustments, Postural Responses, Sensory Orientation, and Stability in Gait improved significantly, while Stability Limits did not. At 12 months postoperatively, ODI decreased by 19.1%, ZCQ symptom and function scores improved by 0.8 and 0.9 points, respectively, and VAS scores improved by 17.1 mm for low back pain, 26.5 mm for lower limb pain, and 19.5 mm for numbness, all showing marked improvements from baseline. MFES also increased significantly postoperatively. The Brief BESTest score correlated significantly with MFES and ZCQ-PFS at baseline, and with ODI, ZCQ, and VAS scores at 12 months. **Conclusions:** Balance ability in LSS patients improved after surgery, as measured by the Brief BESTest, with clinically meaningful changes maintained for 12 months. Improvements in balance were significantly associated with reductions in pain, disability, and fear of falling, suggesting the Brief BESTest is a comprehensive indicator of postoperative recovery.

## 1. Introduction

Lumbar spinal stenosis (LSS) is a degenerative condition based on the compression of neural elements due to age-related changes, which include hypertrophy of the facet joints, thickening of the ligaments, and the intervertebral discs bulging [1]. This condition leads to symptoms including lower limb pain, numbness, and neurogenic intermittent claudication, and is recognized as a significant cause of mobility impairment in the elderly population [2]. Patients with LSS often experience gait disturbances due to sensory deficits in the lower limbs and impaired balance function, which increases the risk of falls and contributes to a decrease in independence in activities of daily living (ADL) [3]. Several studies have demonstrated that balance function is correlated with patient-reported outcomes, including the Oswestry Disability Index (ODI) and Visual Analog Scales (VAS) for leg and back pain, as well as numbness [4,5]. In particular, lower balance performance has been associated with greater disability and pain, highlighting the clinical significance of incorporating balance assessment into patient outcome evaluations. These findings support the notion that balance dysfunction is not only a biomechanical issue but also an important determinant of quality of life in LSS patients. Therefore, assessing balance function is essential in evaluating treatment outcomes in patients with LSS.

Traditionally, balance assessment in LSS patients has relied on simple functional tests such as the Timed Up and Go (TUG) test and One-Leg Standing (OLS) test [6]. However, these tools assess balance from a unidimensional perspective and don’t fully represent the intricate nature of postural control [7]. Furthermore, previous studies using accelerometry during gait or center of pressure (COP) measurements have reported improvements in dynamic and static balance from 6 to 12 months after surgery in patients with LSS [8,9]. However, these assessments evaluate particular aspects of balance and may not reflect broader dimensions such as postural responses, anticipatory adjustments, and sensory integration.

Recently, the Balance Evaluation Systems Test (BESTest) was developed to provide a comprehensive assessment of balance function [10]. The BESTest is unique in that it structurally evaluates balance across six domains: (1) biomechanical constraints, (2) stability limits, (3) anticipatory postural adjustments, (4) postural responses, (5) sensory orientation, and (6) stability in gait. Its usefulness has been demonstrated in both neurological and musculoskeletal populations [11,12]. A shortened version, the Brief BESTest, was later introduced to facilitate clinical use. Despite taking only about eight minutes to administer, it allows for a multidimensional assessment of balance, making it highly practical in clinical settings [13].

Therefore, this study aimed to comprehensively assess longitudinal changes in balance function using the Brief BESTest in patients with LSS from preoperative status to 12 months after surgery, and to clarify the relationship between balance performance and patient-reported outcome measures (PROMs) at each time point.

## 2. Materials and Methods

**Figure 1:**
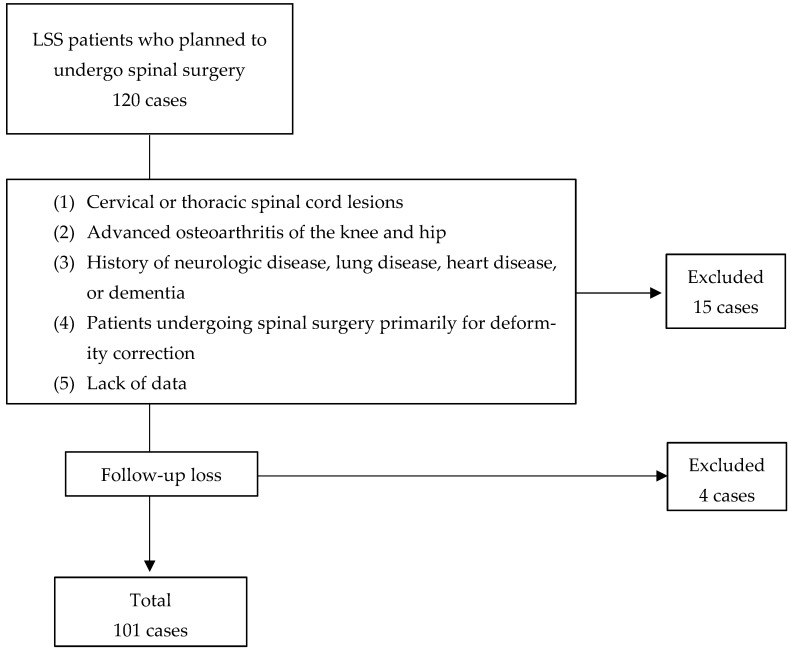
Patient selection flow diagram.

This was a prospective cohort study conducted at a single institution. The study was approved by the Ethics Committee of Okayama Rosai Hospital (approval number: 328; approval date: 31 May 2022). All patients agreed to participate after being fully informed and signing a consent form after receiving a detailed explanation of the study’s purpose and procedures. Between June 2022 and April 2023, a total of 120 patients who underwent spinal surgery for LSS were screened for eligibility. The inclusion criteria were: (1) age ≥ 60 years; (2) decompression or fusion surgery for LSS; (3) ability to walk more than 10 m; and (4) ability to provide informed consent. The exclusion criteria were: (1) myelopathy; (2) advanced osteoarthritis of the hip or knee; (3) a history of neurologic, pulmonary, cardiac diseases, or dementia; (4) patients with severe deformity; and (5) insufficient data. Based on these criteria, 101 patients were included in the final analysis (Figure 1).

The Brief BESTest was administered by physical therapists preoperatively and at 6 months and 12 months postoperatively. Other outcome measures, including the Oswestry Disability Index (ODI), the Modified Falls Efficacy Scale (MFES), the Zurich Claudication Questionnaire (ZCQ), and pain assessments using the Visual Analog Scale (VAS) for lumbago, leg pain, and numbness, were collected preoperatively and at 6 and 12 months postoperatively via self-administered questionnaires.

### 2.1. Patient and Surgical Factors

Patient factors included age, sex, and body mass index (BMI). Surgical factors included the type of surgical procedure, the number of operated intervertebral levels, the specific surgical levels (e.g., L1/2 to L5/S1), operative time, and intraoperative blood loss.

### 2.2. Brief Balance Evaluation Systems Test (BESTest)

**Figure 2:**
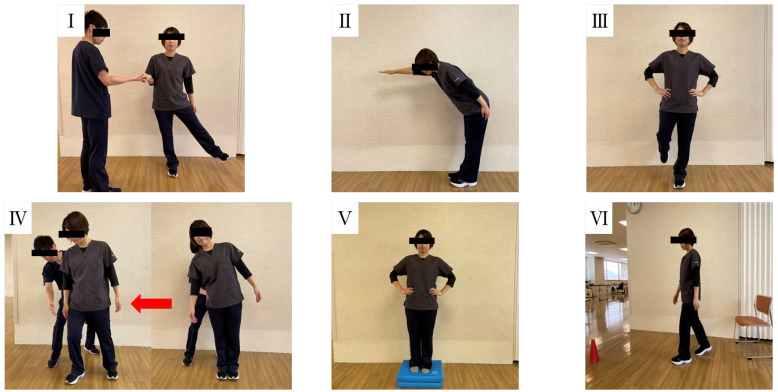
Representative tasks of each domain in the Brief BESTest. (**I**). Hip/trunk lateral strength, (**II**). Functional reach forward, (**III**). Stand on one leg (left and right), (**IV**). Compensatory stepping correction, lateral (left and right), (**V**). Stance on foam, eyes closed, (**VI**). Timed “Get Up and Go” test.

Balance performance was evaluated using the Brief Balance Evaluation Systems Test (Brief BESTest), a shortened version of the original BESTest [10]. The Brief BESTest consists of 6 sections (biomechanical constraints, stability limits, anticipatory postural adjustments, postural responses, sensory orientation, and stability in gait), with a total of 8 items scored on a 4-point ordinal scale (0–3). The total score ranges from 0 to 24, with higher scores indicating better balance function [13] (Table 1, Figure 2).

### 2.3. Oswestry Disability Index (ODI)

The Oswestry Disability Index (ODI) was used to assess the severity of disability related to low back pain [15]. The questionnaire includes 10 sections covering daily activities such as personal care, lifting, walking, and social life. Each section is measured on a scale from 0 to 5, with total scores converted to a percentage ranging from 0% (no disability) to 100% (maximum disability). Higher scores reflect greater functional impairment.

### 2.4. Modified Falls Efficacy Scale (MFES)

Fear of falling in daily life was assessed using the Modified Falls Efficacy Scale (MFES), which evaluates an individual’s confidence in performing 14 common indoor and outdoor activities without falling [16]. Each item is rated on a scale from 0 (not confident at all) to 10 (completely confident), and the mean score is used for analysis. A lower score indicates reduced confidence in maintaining balance during activities.

### 2.5. Zurich Claudication Questionnaire (ZCQ)

The Zurich Claudication Questionnaire (ZCQ), a disease-specific instrument for lumbar spinal stenosis, was used to assess symptom severity, physical performance, and patient satisfaction [17]. It comprises three domains: the Symptom Severity Scale (SSS, comprising seven items), the Physical Function Scale (PFS, comprising five items), and the Satisfaction Scale (SFS, comprising six items). Each item is measured on a scale from 1 (best) to 5 (worst) for SSS and PFS, and from 1 (most satisfied) to 4 (least satisfied) for SFS. Domain scores are calculated as the mean of item scores in each section. Higher SSS and PFS scores indicate more severe symptoms and functional limitation, while lower SFS scores reflect greater satisfaction with treatment.

### 2.6. Pain Assessment

Pain was assessed using the Visual Analog Scale (VAS), with scores ranging from 0 to 100 mm, where 0 indicates “no pain or symptoms” and 100 indicates “worst imaginable pain or symptoms.” Three specific symptom categories were evaluated: low back pain (LBP), lower limb pain (LLP), and lower limb numbness (LLN), including the buttocks and legs. Patients were instructed to mark the intensity of each symptom on a 100 mm horizontal line, and the distance from the left end to the mark was measured in millimeters to determine the VAS score for each symptom.

### 2.7. Statistical Analysis

Changes over time (from preoperative to 12 months postoperative) in the total Brief BESTest score, individual domain scores of the Brief BESTest, ODI, MFES, ZCQ, and VAS were compared using the Friedman test. Post hoc pairwise comparisons were performed with Bonferroni correction. Spearman’s rank correlation coefficients were calculated to assess the relationships between the Brief BESTest and ODI, MFES, ZCQ, and VAS at each time point. Statistical analyses were conducted using EZR (version 1.61, Saitama Medical Center, Jichi Medical University), which is a graphical user interface for R [18]. Spearman’s correlation coefficients (ρ) were interpreted as follows: 0.1 to <0.4 as weak, 0.4 to <0.7 as moderate, 0.7 to <0.9 as strong, and ≥0.9 as very strong [19]. A *p*-value of less than 0.05 was considered statistically significant.

## 3. Results

### 3.1. Patient and Surgical Factors

A total of 101 patients were included in the analysis. The mean age was 74.9 ± 6.9 years, and 60 patients (59.4%) were male. The mean body mass index (BMI) was 24.6 ± 3.4 kg/m^2^. Regarding surgical procedures, 64 patients underwent decompression surgery, while 37 underwent fusion surgery. The number of surgical segments was one in 65 patients and two or more in 36 patients (Table 2).

### 3.2. Changes in Brief BESTest

Compared to the preoperative score of 13.3 ± 5.3, the total Brief BESTest score significantly increased to 16.1 ± 5.1 at 6 months postoperatively (*p* < 0.01) and remained significantly higher at 12 months (16.0 ± 5.1, *p* < 0.01). For the individual domains, the biomechanical constraints domain improved significantly from 1.3 ± 1.1 at baseline to 1.6 ± 1.1 at 6 months (*p* < 0.01) and was still significantly higher at 12 months (1.6 ± 1.2, *p* < 0.05). No significant changes were observed in the stability limits domain, which remained at 2.1 ± 0.5 preoperatively and postoperatively.

The score for anticipatory postural adjustments significantly improved from 3.5 ± 2.1 preoperatively to 4.6 ± 1.6 at 6 months postoperatively (*p* < 0.01), and remained significantly higher at 12 months (4.4 ± 1.8, *p* < 0.05 compared to baseline). Postural responses also showed a significant improvement, increasing from 2.8 ± 1.7 preoperatively to 3.4 ± 1.7 at 6 months (*p* < 0.01) and remaining significantly elevated at 12 months (3.2 ± 1.8, *p* < 0.05). The sensory orientation domain did not show a significant difference at 6 months but did show a significant improvement from 1.6 ± 1.0 preoperatively to 2.0 ± 1.0 at 12 months (*p* < 0.01). The stability in gait domain significantly increased from 2.1 ± 1.0 to 2.7 ± 0.6 at 6 months and remained at 2.7 ± 0.5 at 12 months (*p* < 0.01 for both time points compared to baseline) (Table 3).

### 3.3. Changes in ODI

The Oswestry Disability Index (ODI) showed a significant decrease from 42.2 ± 15.9% preoperatively to 19.9 ± 17.6% at 6 months and 23.1 ± 17.5% at 12 months postoperatively (both *p* < 0.01) (Table 3).

### 3.4. Changes in MFES

The MFES significantly increased from 97.8 ± 32.6 points preoperatively to 115.6 ± 28.8 at 6 months and 115.9 ± 27.1 at 12 months postoperatively (both *p* < 0.01) (Table 3).

### 3.5. Changes in ZCQ

The Zurich Claudication Questionnaire (ZCQ) Symptom Severity Scale (SSS) score improved significantly from 3.1 ± 0.5 to 2.2 ± 0.7 at 6 months and 2.3 ± 1.3 at 12 months postoperatively (both *p* < 0.01). The Physical Function Scale (PFS) also improved from 2.7 ± 0.6 preoperatively to 1.8 ± 0.6 at both 6 and 12 months (*p* < 0.01). The Satisfaction Scale (SFS) at 12 months was 2.0 ± 0.8, with a mild but statistically significant improvement from 6 months (1.8 ± 0.6, *p* < 0.05). All time point outcomes for ZCQ are shown in Table 3.

### 3.6. Changes in Pain and Numbness

LBP VAS scores significantly decreased from 36.3 ± 30.1 mm preoperatively to 17.5 ± 23.1 mm at 6 months and 19.2 ± 23.6 mm at 12 months (both *p* < 0.01). LLP VAS also showed a significant reduction to 21.9 ± 26.6 mm at 6 months and 24.0 ± 29.2 mm at 12 months compared to the preoperative value of 50.5 ± 30.1 mm (both *p* < 0.01). Similarly, LLN VAS significantly improved from 45.3 ± 33.2 mm preoperatively to 18.7 ± 26.3 mm at 6 months and 25.8 ± 32.3 mm at 12 months (both *p* < 0.01).

### 3.7. Correlation Between Brief BESTest and Patient-Reported Outcome Measures

At the preoperative time point, the Brief BESTest total score was significantly correlated with the MFES (ρ = 0.439, *p* < 0.001) and the ZCQ Physical Function Scale (PFS) (ρ = −0.419, *p* < 0.001). No significant correlations were found with ODI, ZCQ Symptom Severity Scale (SSS), or any VAS scores. At 6 months postoperatively, the Brief BESTest score remained significantly correlated with MFES (ρ = 0.409, *p* < 0.001). Significant negative correlations were also observed with ZCQ SSS (ρ = −0.346, *p* = 0.014), ZCQ PFS (ρ = −0.313, *p* = 0.025), ZCQ Satisfaction Scale (SFS) (ρ = −0.409, *p* = 0.004), and LBP VAS (ρ = −0.299, *p* = 0.010). Correlations with ODI, LLP VAS, and LLN VAS were not statistically significant.

At 12 months postoperatively, significant correlations were again found between the Brief BESTest and MFES (ρ = 0.451, *p* < 0.001), ODI (ρ = −0.442, *p* = 0.027), ZCQ SSS (ρ = −0.481, *p* < 0.001), ZCQ PFS (ρ = −0.295, *p* = 0.013), and ZCQ SFS (ρ = −0.299, *p* = 0.013). In addition, the Brief BESTest score showed significant negative correlations with LBP VAS (ρ = −0.331, *p* < 0.001), LLP VAS (ρ = −0.275, *p* = 0.006), and LLN VAS (ρ = −0.206, *p* = 0.042).

All time point correlations between the Brief BESTest and Patient-Reported Outcomes are shown in Table 4.

## 4. Discussion

Patients with lumbar spinal stenosis (LSS) often experience impaired balance and an increased risk of falls due to reduced lumbar motion [20,21]. These functional limitations can negatively affect postural control and mobility, making balance assessment and rehabilitation essential components of postoperative management in this population. The Balance Evaluation Systems Test (BESTest) is a comprehensive clinical tool designed to assess multiple domains of balance control and is associated with fall risk in various patient populations [7,22]. Shorter versions of the BESTest, such as the Mini-BESTest and Brief BESTest, have been developed to facilitate clinical application with reduced administration time while maintaining multidimensional balance assessment capability [13]. Among these, the Brief BESTest offers a particularly efficient assessment format, making it suitable for use in busy clinical settings. Recent studies have demonstrated associations between balance ability, as assessed using the Mini-BESTest, and gait ability in patients with LSS [23]. However, to date, no studies have examined postoperative changes in balance performance using the Brief BESTest in patients with LSS. The present study is the first to evaluate postoperative changes in balance ability using the Brief BESTest in patients with LSS and to investigate its relationships with patient-reported outcome measures (PROMs), including the Oswestry Disability Index (ODI), Modified Falls Efficacy Scale (MFES), Zurich Claudication Questionnaire (ZCQ), and Visual Analog Scales (VAS) for pain and numbness.

In this study, significant improvements in the Brief BESTest were observed at both 6 and 12 months postoperatively compared to preoperative scores. Notably, the increase in total scores suggests a comprehensive enhancement of balance function, indicating that surgical intervention can help restore multifaceted balance abilities in individuals with LSS. Domain-specific analyses revealed that improvements in biomechanical constraints and anticipatory postural adjustments may reflect enhanced muscle strength and joint range of motion, likely attributable to the effects of postoperative rehabilitation. These two domains are known to involve the function of the hip abductor muscles [24]. Since the most frequently affected level in LSS is L4/5, and previous studies have reported reduced gluteus medius strength in such cases [25], it is reasonable to consider that postoperative improvements in gluteus medius function contributed to gains in these balance components. Postural responses improved bilaterally after surgery in this study, suggesting a recovery of the ability to maintain postural control in response to external perturbations. This result may be due to the surgical improvement of lower limb pain and numbness.

Sensory orientation was not improved at 6 months; however, it improved at 12 months postoperatively. This domain needs to improve for a longer time compared with other domains, which includes tasks such as standing on an unstable foam surface with eyes closed—an act that heavily relies on proprioceptive input [26]. Previous research has indicated that proprioception may improve following LSS surgery [27], and our results support this, suggesting that recovery of proprioceptive function may occur more gradually compared to other balance domains. In particular, the test item for stability in gait is the Timed Up and Go (TUG) test, which showed improvement across all time points. The TUG is a reliable and valid measure of functional mobility and is associated with locomotor dysfunction in patients with degenerative spinal disorders [28,29]. In contrast, no significant change was observed in the stability limits domain, which is assessed using the Functional Reach Test (FRT). Previous studies have shown that FRT scores are lower in patients with LSS than in healthy individuals and are associated with spinal dysfunction and quality of life [30]. FRT performance is also known to be influenced by spinal flexibility [31]. In the present study, the majority of cases underwent decompression surgery, or one-level surgical fixation, which may have minimized the impact of surgery on spinal flexibility.

Halvarsson et al. developed a specific progressive balance training program incorporating dual- and multi-task exercises based on the six systems of the BESTest for older adults [32]. These exercises may also contribute to improving balance performance in patients with LSS, given the similarities in age-related balance impairments. Hence, future interventional studies are warranted to investigate the effectiveness of such system-based balance training programs specifically tailored for the LSS population.

Concerning PROMs, significant improvements were observed in ODI, MFES, ZCQ, and VAS scores at both 6 and 12 months postoperatively, confirming symptom relief and functional recovery. At 12 months, the average changes from baseline were 19.1% for ODI, 0.8 points for ZCQ SSS, 0.9 points for ZCQ PFS, 17.1 mm for LBP, 26.5 mm for LLP, and 19.5 mm for LLN. These changes exceeded the minimal clinically important differences previously reported for each PROM [33,34], indicating clinically meaningful improvement. Importantly, to our knowledge, this is the first study to longitudinally assess fall-related self-efficacy using the MFES in patients with lumbar spinal stenosis. Our findings suggest that spine surgery contributes not only to physical recovery but also to psychological aspects such as confidence in avoiding falls, which is particularly relevant in an aging population. In terms of treatment satisfaction, the ZCQ SFS scores were 1.8 at 6 months and 2.0 at 12 months, indicating high levels of postoperative satisfaction. A score of ≤2.5 on the SFS is generally considered the threshold for treatment satisfaction [35].

Correlations between the Brief BESTest and PROMs revealed significant associations preoperatively with MFES and ZCQ-PFS. At 6 and 12 months postoperatively, stronger and broader correlations were identified, particularly at 12 months, where significant correlations were noted with ODI, ZCQ SSS, PFS, SFS, and all VAS subscales (LBP, LLP, LLN). These findings suggest that the Brief BESTest is not merely a balance assessment tool, but may also reflect broader aspects of patients’ physical function, symptoms, and satisfaction after surgery. Notably, Thornes et al. reported that four domains of the Mini-BESTest—anticipatory postural adjustments, postural responses, sensory orientation, and stability in gait—were closely associated with ZCQ PFS scores [23], which is consistent with our findings. Therefore, improving balance function after surgery may contribute to enhanced health-related quality of life and patient satisfaction in individuals with LSS.

The MFES was significantly correlated with the Brief BESTest at all time points from preoperative to 12 months, suggesting that improvements in balance ability may enhance fall-related self-efficacy. This highlights the possibility that balance recovery contributes not only to physical but also to psychological aspects of postoperative rehabilitation.

This study has several limitations. First, although this was a prospective study, it was conducted at a single institution with a limited sample size, which may affect the generalizability of the findings. Second, the follow-up period of this study is relatively short. Third, while balance function was assessed using the Brief BESTest, we did not evaluate objective outcomes such as fall incidence or physical activity levels.

## 5. Conclusions

This study is the first to longitudinally evaluate postoperative changes in balance ability using the Brief BESTest in patients with LSS. Significant improvements were observed in the total score, as well as in specific domains, including biomechanical constraints, anticipatory postural adjustments, postural responses, and stability in gait, all of which showed improvement 6 months after surgery. In contrast, sensory orientation showed delayed recovery, with significant improvement observed only at 12 months. These findings suggest that while most aspects of balance function recover relatively quickly following surgical intervention and rehabilitation, proprioceptive-related balance function may require a longer recovery period. The Brief BESTest proved to be a clinically useful tool for capturing these domain-specific recovery patterns, supporting its use for comprehensive postoperative assessment and guiding targeted balance rehabilitation in patients with LSS.

## Figures and Tables

**Table 1:** Domains and items of the Brief BESTest [14].

Domain No.	Domain Name	Task Description
(I)	Biomechanical constraints	Hip/trunk lateral strength
(II)	Stability limits	Functional reach forward
(III)	Anticipatory postural adjustments	Stand on one leg (left and right)
(IV)	Postural responses	Compensatory stepping correction, lateral (left and right)
(V)	Sensory orientation	Stance on foam, eyes closed
(VI)	Stability in gait	Timed “Get Up and Go” test

**Table 2:** Patient demographics.

Mean ± SD; *n*	
Age (years)	74.9 ± 6.9
Sex	
Male	60
Female	41
Body Mass Index (kg/m^2^)	24.6 ± 3.4
Surgical Type	
Decompression	64
Fusion	37
Surgical segment	
1	65
≥2	36
Surgical levels	
L1/2	2
L2/3	8
L3/4	33
L4/5	79
L5/S	14
Operative time (min)	112.7 ± 45.9
Blood loss (mL)	120.8 ± 121.4

Data are presented as mean ± standard deviation (SD). “*n*” indicates the number of participants.

**Table 3:** Changes in balance and patient-reported outcomes over time.

	Pre-Op	6 M Post-Op	12 M Post-Op
Brief BESTest (Total Score)	13.3 ± 5.3	16.1 ± 5.1 **	16.0 ± 5.1 **
I. Biomechanical constraints (pt)	1.3 ± 1.1	1.6 ± 1.1 **	1.6 ± 1.2 *
II. Stability limits (pt)	2.1 ± 0.5	2.1 ± 0.4	2.1 ± 0.4
III. Anticipatory postural adjustments (pt)	3.5 ± 2.1	4.6 ± 1.6 **	4.4 ± 1.8 *
IV. Postural responses (pt)	2.8 ± 1.7	3.4 ± 1.7 **	3.2 ± 1.8 *
V. Sensory orientation (pt)	1.6 ± 1.0	1.9 ± 1.1	2.0 ± 1.0 **
VI. Stability in gait (pt)	2.1 ± 1.0	2.7 ± 0.6 **	2.7 ± 0.5 **
LBP VAS (0–100)	36.3 ± 30.1	17.5 ± 23.1 **	19.2 ± 23.6 **
LLP VAS (0–100)	50.5 ± 30.1	21.9 ± 26.6 **	24.0 ± 29.2 **
LLN VAS (0–100)	45.3 ± 33.2	18.7 ± 26.3 **	25.8 ± 32.3 **
MFES (pt)	97.8 ± 32.6	115.6 ± 28.8 **	115.9 ± 27.1 **
ODI (%)	42.2 ± 15.9	19.9 ± 17.6 **	23.1 ± 17.5 **
ZCQ SSS (pt)	3.1 ± 0.5	2.2 ± 0.7 **	2.3 ± 1.3 **
ZCQ PFS (pt)	2.7 ± 0.6	1.8 ± 0.6 **	1.8 ± 0.6 **
ZCQ SFS (pt)	NA	1.8 ± 0.6	2.0 ± 0.8

Data are presented as mean ± standard deviation (SD). * *p* < 0.05, ** *p* < 0.01 compared with preoperative value. Abbreviations: ODI, Oswestry Disability Index; MFES, Modified Falls Efficacy Scale; ZCQ, Zurich Claudication Questionnaire; SSS, Symptom Severity Scale; PFS, Physical Function Scale; SFS, Satisfaction Scale; VAS, Visual Analog Scale; LBP, low back pain; LLP, lower limb pain; LLN, lower limb numbness; 6 M, 6 months; 12 M, 12 months; Post-Op, postoperative; pt, point; NA, not assessed.

**Table 4:** Spearman’s rank correlation coefficients between the Brief BESTest and patient-reported outcome measures at preoperative, 6-month, and 12-month.

Time Point	Outcome Measure	Spearman’s ρ	*p*-Value
Preoperative	MFES	0.439	<0.001
	ODI	−0.301	0.163
	ZCQ SSS	−0.172	0.153
	ZCQ PFS	−0.419	<0.001
	LBP VAS	−0.012	0.906
	LLP VAS	−0.141	0.172
	LLN VAS	0.013	0.902
6 months postoperatively	MFES	0.409	<0.001
	ODI	−0.336	0.126
	ZCQ SSS	−0.346	0.014
	ZCQ PFS	−0.313	0.025
	ZCQ SFS	−0.409	0.004
	LBP VAS	−0.299	0.010
	LLP VAS	−0.202	0.086
	LLN VAS	−0.228	0.053
12 months postoperatively	MFES	0.451	<0.001
	ODI	−0.442	0.027
	ZCQ SSS	−0.481	<0.001
	ZCQ PFS	−0.295	0.013
	ZCQ SFS	−0.299	0.013
	LBP VAS	−0.331	<0.001
	LLP VAS	−0.275	0.006
	LLN VAS	−0.206	0.042

Abbreviations: ODI, Oswestry Disability Index; MFES, Modified Falls Efficacy Scale; ZCQ, Zurich Claudication Questionnaire; SSS, Symptom Severity Scale; PFS, Physical Function Scale; SFS, Satisfaction Scale; VAS, Visual Analog Scale; LBP, low back pain; LLP, lower limb pain; LLN, lower limb numbness.

## Data Availability

The data presented in this study are available on request from the corresponding author due to ethical reasons.
